# Benchmarking single-sample gene set scoring methods for application in precision medicine

**DOI:** 10.1093/bib/bbaf684

**Published:** 2025-12-17

**Authors:** Daniel Toro-Domínguez, Chang Wang, Iván Ellson-Lancho, Jordi Martorell-Marugán, Raúl López-Domínguez, Pedro Carmona-Sáez, Marta E Alarcón-Riquelme, Frédéric Baribaud

**Affiliations:** Unit of Inflammatory Diseases, Department of Environmental Medicine, Karolinska Institute, Nobel väg 13, Solna 171 67, Sweden; Bristol Myers Squibb Research & Early Development, 3551 Lawrenceville Road, Princeton 08648, New Jersey, United States; Bristol Myers Squibb Research & Early Development, 3551 Lawrenceville Road, Princeton 08648, New Jersey, United States; GENYO. Centre for Genomics and Oncological Research: Pfizer, University of Granada, Andalusian Regional Government, PTS Granada, Avenida de la Ilustración 114, Granada 18016, Spain; GENYO. Centre for Genomics and Oncological Research: Pfizer, University of Granada, Andalusian Regional Government, PTS Granada, Avenida de la Ilustración 114, Granada 18016, Spain; Fundación para la Investigación Biosanitaria de Andalucía Oriental-Alejandro Otero (FIBAO), Avenida de Madrid 15, Granada 18012, Spain; GENYO. Centre for Genomics and Oncological Research: Pfizer, University of Granada, Andalusian Regional Government, PTS Granada, Avenida de la Ilustración 114, Granada 18016, Spain; GENYO. Centre for Genomics and Oncological Research: Pfizer, University of Granada, Andalusian Regional Government, PTS Granada, Avenida de la Ilustración 114, Granada 18016, Spain; Department of Statistics, University of Granada, Avenida de Fuentenueva s/n, Granada 18071, Spain; Unit of Inflammatory Diseases, Department of Environmental Medicine, Karolinska Institute, Nobel väg 13, Solna 171 67, Sweden; GENYO. Centre for Genomics and Oncological Research: Pfizer, University of Granada, Andalusian Regional Government, PTS Granada, Avenida de la Ilustración 114, Granada 18016, Spain; Bristol Myers Squibb Research & Early Development, 3551 Lawrenceville Road, Princeton 08648, New Jersey, United States

**Keywords:** single-sample, gene set scoring, transcriptomics, patient stratification, predictive modeling, data integration, precision medicine

## Abstract

Gene set-based single-sample scoring methods are promising to elucidate patient level disease heterogeneity and enable functional interpretation of molecular data for precision medicine approaches. Despite the availability of numerous algorithms, their performance under different scenarios and for downstream applications for precision medicine approaches has not been systematically evaluated. In this study, we conducted a comprehensive survey of an exhaustive list of single-sample scoring methods to assess their stability and reproducibility performances under commo scenarios which include limitations of input data or data integration across studies. We also evaluated their performances for downstream patient stratification and clinical association analyses, as well as predictive modeling of disease states. The in-depth characterization of these scoring methods highlights the importance for a rational design of analysis strategies and provides fundamental insights into method selection under different scenarios or for different applications.

## Introduction

Precision medicine consisting of tailored treatment regimens for each patient is emerging as a promising approach for complex diseases, characterized by heterogeneity in clinical manifestations and treatment responses. The underlying hypothesis is that phenotypic heterogeneity stems from intrinsic molecular heterogeneity in disease pathogenesis [1]. Therefore, a molecular pathology-guided therapeutic design offers a targeted option with increased treatment success [2]. Genetic and immune profiling have been used to support precision medicine. Omics data, particularly transcriptomics, have shown potential for patient stratification and predictive modeling using machine learning (ML). Several studies have demonstrated that molecularly defined patient subgroups also exhibited strong differences in clinical features and treatment responses [3–7].

Among omic-based approaches, transcriptomics has become a key method to capture molecular heterogeneity across patients and uncover complex disease pathobiology. It has generated a growing body of public data, offering opportunities for integrative analyses. However, integrating transcriptomic data across multiple studies remains challenging due to intrinsic, extrinsic, and technical confounders. Batch effects -within a study (technical variation) or between studies (technical and biological variation)- are a major concern, often without effective mitigation [8]. Moreover, the high dimensionality of transcriptomic data imposes challenges to computational resources and downstream biological interpretation.

Gene set-based single-sample scoring has emerged as a potential solution to these challenges, preserving individual-level heterogeneity while reducing dimensionality by transforming gene-level data into pathway-level activities. This facilitates interpretation of biological processes underlying disease. As scoring is performed per sample, it captures individual variation—crucial for characterizing heterogeneity and stratifying patients. Numerous algorithms exist, implementing different strategies for score calculation. For example, methods such as Gene Set Variation Analysis (GSVA) [9], variations of Gene Set Enrichment Analysis (GSEA) such as single-sample Gene Set Enrichment Analysis (ssGSEA) and Fast Gene Set Enrichment Analysis (FGSEA) [10, 11], and singscore [12], employ rank-based comparisons, though their ranking mechanisms differ. In contrast, M-scores [5], Pathway-Level Analysis of Gene Expression (PLAGE) [13] and combined Z-score [14] compare expression distributions -either between query and reference samples (M-scores), via singular value decomposition (PLAGE) or by aggregating z-scores (Z-score). A variety of methods have also been adopted for score calculation, ranging from sum- or mean-based aggregation methods such as Weighted Sum (WSUM) and Weighted Mean (WMEAN) [15], to Area Under the Curve (AUC) analysis implemented in AUCell [16], one-tailed Fisher’s exact test in Over-Representation Analysis (ORA) [15], decision trees in Univariate Decision Tree (UDT) [15] and Multivariate Decision Tree (MDT) [15], and linear modeling in Univariate Linear Model (ULM) [15] and Multivariate Linear Model (MLM) [15].

Despite the availability of these methods, there have been limited efforts to systemically benchmark their performance. Understanding of their downstream use in ML or clustering remains scarce. Prior benchmarks focus mostly on computational aspects—run-time, score stability, and recall [12, 15]. Yet common practical challenges, such as differences in input quality, reference gene sets, or batch effects, are rarely assessed. Moreover, how these methods perform in downstream modeling and clustering remain unclear, posing a barrier to their broader use in precision medicine.

We present a comprehensive evaluation of 18 single-sample scoring methods under varied scenarios reflecting challenges frequently encountered in gene expression data and reference gene set databases, and when integrating data from multiple studies. We also assess their downstream application in Systemic Lupus Erythematosus (SLE), a highly heterogeneous autoimmune disease, providing a case study on how single-sample scoring may inform personalized treatment decisions.

## Material and methods

### Datasets

Five transcriptomic datasets from SLE studies were used for benchmarking. One, from the PRECISESADS project [17], includes 376 SLE patients and 263 normal healthy volunteers (NHVs). It was used to assess runtime performance, score similarities, and the impact of various factors associated with the input data on score stability. Clinical and serological data were used to determine the association between molecular scores and clinical observations.

To test batch effect mitigation and data integration across studies, four public datasets were retrieved from the National Centre for Biotechnology Information Gene Expression Omnibus database [18]: GSE65391, GSE99967, GSE72326, and GSE45291 [19–21]. After data pre-processing, samples with active proliferative lupus nephritis (pLN, biopsy-confirmed <1 year), were retained as pLN samples, and samples from patients without renal involvement were labeled as NoLN (Non-Lupus Nephritis patients). In total, 179 NHV, 138 pLN, and 214 NoLN samples from those four datasets were used.

An SLE-Diseaseome [22] containing 5212 curated SLE-related gene sets was used for scoring, capturing a broad spectrum of molecular functions behind pathology [22].

### Data pre-processing

Each dataset was processed independently in a platform-specific manner [23]. For RNAseq data, transcripts with at least 10 counts in more than 10% of the samples were retained. The counts were normalized using the trimmed mean of M-values method from the NOISeq R package [24]. Transcripts mapped to the same gene were merged by median expression, followed by log2-transformation. For microarray data, the probe expression matrix was annotated to gene symbols in the same way as RNAseq data, followed by log2-transformation. Lastly, within the gene expression matrixes for both platforms, genes with near to zero variance per group (NHV, pLN, and NoLN) were removed using the caret R package [25].

### Single-sample scoring

Eighteen single-sample scoring methods were benchmarked. These included M-scores, GSVA, ssGSEA, singscore, Z-score, PLAGE, AUCell, MDT, MLM, ORA, UDT, ULM, FGSEA, WMEAN, and WSUM. For WMEAN, WSUM, and FGSEA, normalized variants were generated (norm_WMEAN and norm_WSUM) or with permutation-based correction (norm_FGSEA) [26]. Scores were computed using the *getScores* function in the pathMED R package [27], discarding gene sets with <3 genes. Permutations were set to 100 for permutation-based metrics. For network-based methods (AUCell, MDT, MLM, ULM, UDT, WMEAN, norm_WMEAN, WSUM, and norm_WSUM), gene weights were set to 1 [15].

### Runtime performance and score similarity

The runtime performance was assessed by evaluating three factors: the sample size of gene expression data, the number of gene sets and the average number of genes per gene set in the gene set database. It is worth noting that runtime is hardware- and implementation-setting-dependent, and therefore differs from computational complexity due to the underlying algorithm. Thus, this test compared the relative execution times of different methods under standardized computing conditions. First, to evaluate the impact of sample size, variable numbers of samples, ranging from 3 to 600, were randomly selected from the PRECISESADS dataset and scored over 100 randomly selected gene sets from the SLE-Diseaseome [22]. Second, to evaluate the impact of the number of gene sets, variable numbers of gene sets, ranging from 1 to 1500, were randomly selected and used to score a set of 50 randomly selected samples. Lastly, to evaluate the impact of the average size of gene sets, 50 gene sets assembled from a variable number of genes, ranging from 5 to 600 per gene set, were used to score a set of 50 samples. All analyses were conducted in a computing environment running Ubuntu 18.04.6 LTS (x86_64), with R version 4.3.1, 64 GiB of RAM, and 32 CPU cores. A preliminary analysis with 250 samples and 500 gene sets was performed to optimize the parameters of the getScores function for each approach. Parallel computing was enabled within getScores function only for M-scores, MDT, UDT, and ULM to achieve their optimal computing capacity, allowing objective comparison with other methods that perform best without parallel computing. It is worth noting that some methods, such as GSVA and functions in the decoupleR package, have their own internal parallelization and thus were run without additional parallelization through getScores.

Score similarities across methods were assessed on the PRECISESADS dataset by pairwise comparisons on Pearson correlation coefficients across samples. Mean correlation across samples for each comparison was used to determine score similarity.

### Impact of sample size on score stability

Sample subsets (ranging from 1% to 90% of PRECISESADS samples) were randomly selected in 50 permutations. Scores were compared to full dataset scores via Pearson correlation and Euclidean distance to determine score stability across different sample sizes.

To further assess the impact on downstream sample clustering analyses, besides the sample subsets described above (target samples), an additional set of 50 randomly selected samples (carrier samples) were also retained for clustering to ensure a sufficiently large sample size for achieving stable clustering results, especially when the number of target samples is small. For each set of target samples, scores were obtained in two ways: (i) calculated using only the set of target samples, and (ii) retrieved from scores calculated using the whole PRECISESADS dataset. In contrast, scores for the carrier samples were always calculated using the whole PRECISESADS dataset. Then, for each sample subset, two types of score matrices were created by joining (using the cbind R function) the carrier samples with the target samples scored either within the target sample set (testing matrix) or with the whole PRECISESADS dataset (reference matrix). Each type of matrices was clustered separately using k-means-based consensus clustering, implemented in the ConsensusClusterPlus function in the ConsensusClusterPlus R package [28] with 250 permutations, together with the Euclidean distance metric and complete linkage, keeping the rest of parameters by default. Failure rate was the fraction of target samples assigned to different clusters between test and reference. Additionally, we tested how the distance metric and clustering method impact the stability of the clustering results with each scoring approach. Specifically, we clustered each score matrix using the entire PRECISESADS cohort using different combinations of clustering method [i.e. k-means-based clustering (KM), partitioning around medoids (PAM), and hierarchical clustering (HC)] and distance metric (i.e. Euclidean distance, Spearman’s and Pearson’s correlation, cosine distance, maximum distance, and Canberra distance), as enabled in the ConsensusClusterPlus function. Then, we calculated the mean proportion of ambiguous clustering across different numbers of clusters (K ranging from 3 to 8) with each combination of clustering method and distance metric.

### Impact of missing genes on score stability

From PRECISESADS, a gradient of missing genes was created by removing a variable percentage of randomly selected genes, ranging from 10% to 90%. Each sampling iteration was repeated 10 times. One hundred randomly selected gene sets with at least three genes were used for scoring to ensure consistency. Score consistency with the whole dataset was evaluated via Pearson correlation and Euclidean distance.

### Impact of the gene set database size on score stability

The order of gene sets in the entire database was first shuffled. Then, a varying number of the top gene sets, ranging from top 1 to top 1000, were retained as subset databases to score samples in the PRECISESADS dataset. Scores of the top 1 gene set were compared across subset databases using Pearson correlation and Euclidean distance. The average correlation coefficient and Euclidean distance were recorded. Ten permutations were performed for each scoring approach independently.

### Sensitivity of scores to inaccurate gene set annotations

Five arbitrary databases (5, 10, 25, 50, or 100 genes per set) of 1000 gene sets each were created by random gene selection to test the sensitivity of scores to false positives. Calculated scores from 100 SLE samples from PRECISESADS cohort were scaled against NHVs. Z-tests assessed whether each SLE sample differed significatively from NHVs. False positive rate (FPR) was the percentage of SLE samples with significantly different scores compared with NHVs.

### Data integration assessment

Four SLE datasets with NHVs, pLN, and NoLN samples were used for data integration testing. Silhouette coefficients were computed using the cluster R package [29] for each method to evaluate the relationship between sample distribution and LN status or the study using: (i) gene set scores, (ii) pre-processed gene expression, and (iii) sample-wise z-scored expression. Principal Component Analysis (PCA) was performed with the prcomp function in the stats package in R to visualize sample distribution.

### Performance of score-based machine learning models across studies

Four SLE datasets were used to test the performance of scores in ML modeling to predict pLN status. GSE65391 and GSE72326 were used for model training and validation, while GSE99967 and GSE45291 were used for testing. For feature selection, a random-effect model-based meta-analysis was performed for each scoring approach using the metaAnalysisDE in the DexMa package [30], selecting the top 150 significant gene sets differentiating pLN from NoLN. Models were trained using the trainModel function in the pathMED package [27] by nested k-fold cross validation [25]. Specifically, the entire dataset for model building was divided into four class-balanced folds with 75% of samples in the training set and 25% in the validation set. Samples from the same patient were assigned to the same set using the pairedColumn parameter. Model hyperparameters were tuned with inner five-fold cross-validation for each training set, repeating three times with random internal initialization and using 80% and 20% of samples as the training and validation sets, respectively. In total, 11 classification algorithms, covering commonly used ML approaches [25], were implemented. Model performances were evaluated in each separated outer test fold, and the algorithm prioritization was based on the average Matthews Correlation Coefficients (MCC) across outer folds to provide an unbiased measurement of accuracy. The entire process was repeated five times using different seeds.

The predictExternal function in the pathMED package was used for independent, retrieving different performance metrics, including MCC, balanced accuracy, precision, recall, specificity, F-score, and negative predictive value. Median and standard deviation of each performance metric were calculated across all the iterations and testing datasets for each scoring approach.

### Association between molecular scores and clinical features

The PRECISESADS patients were stratified based on each scoring method. K-means-based consensus clustering was performed as described above, and enrichment analyses were conducted to test the association between each cluster and 67 different clinical variables, such as demographics, comorbidities, and clinical manifestations, the level of 17 cytokines, and seropositivity of 16 autoantibodies. ANOVA or Kruskal–Wallis was applied for numeric variables, and Fisher’s exact test for categorical ones. Clusters (k = 3 to 7) were tested; the k maximizing significant associations (*P*-value <.05) was used to compare scoring approaches.

## Results

### Runtime performances and results comparison

Depending on the scoring approach and its underlying algorithm, runtime performance varied and was influenced by the sizes of the gene expression matrix and the reference gene set database. To assess the basic performance of available single-sample signature-scoring approaches, we evaluated runtime across varying sample sizes, number of gene sets, and average gene counts per gene set. For each approach, the optimal parallelization configuration enabled in the getScores function in the pathMED R package was selected (Supplementary Fig. S1A). Runtime of FGSEA with score correction (norm_FGSEA) drastically increased when processing larger gene expression data and gene set databases (Supplementary Fig. S1), especially in terms of sample sizes and average number of genes per gene sets. The runtime of UDT also increased with larger sample sizes (Supplementary Fig. S1B) and was more substantially impacted by the number of gene sets (Supplementary Fig. S1C). The runtime of FGSEA showed similar extent of increase with larger sample sizes as UDT but was not impacted as greatly as UDT by larger numbers of gene sets, while being more strongly impacted by the average number of genes per gene sets (Supplementary Fig. S1). M-scores required the longest runtime, regardless of the average number of genes per gene set tested, but were not impacted by increase in the number of genes per gene set as observed with the norm_FGSEA (Supplementary Fig. S1D).

**Figure 1:**
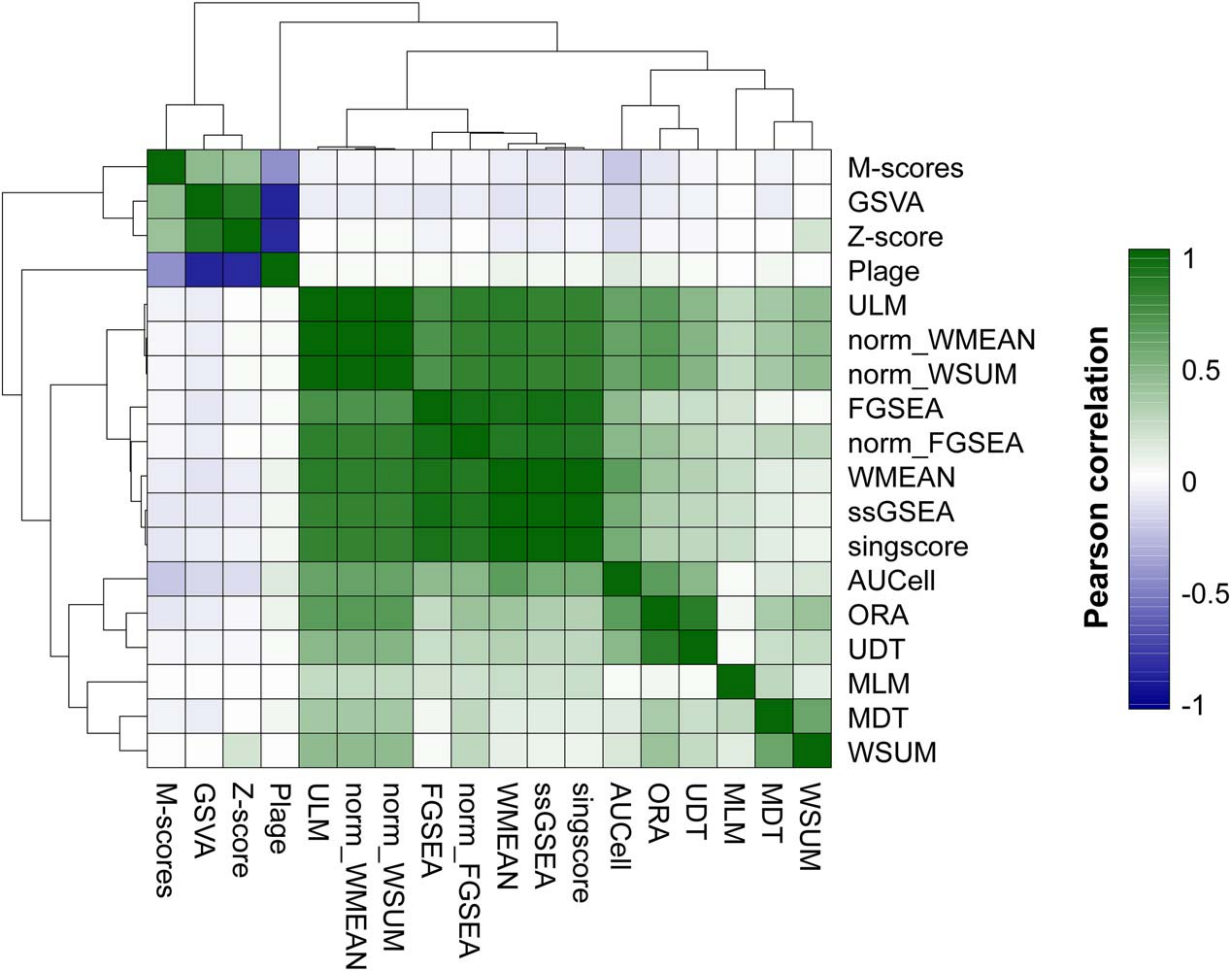
Gene set score correlations across methods. The heatmap shows the average pairwise Pearson correlation coefficients across scores from the PRECISESADS dataset calculated using different scoring methods.

Next, we evaluated score similarity across approaches. As expected, approaches following comparable algorithm principles yielded similar results. For example, several rank-based approaches, such as FGSEA with (norm_FGSEA) or without score correction (FGSEA), ssGSEA, and singscore, resulted in highly similar scores, together with other approaches such as WMEAN, norm_WMEAN, norm_WSUM, and ULM (Fig. 1). Similarly, distribution-based approaches, such as M-scores and Z-score, exhibited similar score profiles forming a group, together with GSVA. But interestingly, those scores showed inverse correlations to scores from PLAGE, potentially due to the arbitrary nature of PLAGE score direction given its singular value decomposition (SVD) based pathway activity assessment [13] (Fig. 1). Two other groups were also observed, including WSUM and MDT, as well as ORA and UDT, although their statistical basis differs, while the rest of approaches such as MLM exhibited relatively unique score profiles (Fig. 1).

### Sample size impacts on score stability

**Figure 2:**
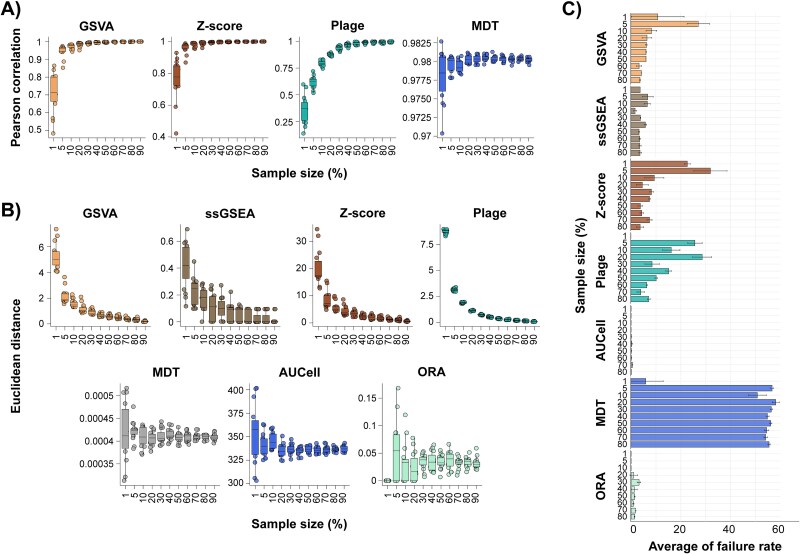
Impact of sample size on score stability. (A) The average Pearson correlation coefficients and (B) the average Euclidean distance scores computed using variable sample sizes of the PRECISESADS SLE dataset, ranging from 1% to 90% of the total number of samples, compared to the scores using the full dataset (*N* = 376). Only the results from scoring approaches with detectable differences are shown. (C) Number of occurrences of target samples assigned to a different cluster when comparing to the results from the testing matrices (subsets of different sample sizes) with the reference matrix (whole dataset), divided by the total number of target samples in any given subset. Colors denote scoring approaches.

Due to differences in methodology across scoring approaches, factors associated with gene expression data and gene set databases, such as inter-sample variability and data quality, may exert profound effects on the stability of scores. Choosing an approach with the best stability according to the features of data is critical to ensure optimal downstream applications and therefore multiple scenarios were explored to assess the effects on score stabilities. The impact of sample size on score stability by taking subsets of the SLE samples from the PRECISESADS cohort and comparing them with scores generated from the full dataset was assessed. Over a wide range of sample sizes tested, including as low as three samples (1% of total), scores from most approaches remained unaffected when considering correlation and Euclidean distance. However, several approaches showed substantially compromised stability with sample sizes below certain limits or over a range of small sample sizes. For example, results from GSVA and Z-score exhibited a marked decrease in correlation coefficients at a sample size below 10% of total (i.e. *n* = 37), while PLAGE was affected below 20% (i.e. *n* = 75) (Fig. 2A). These three approaches, together with ssGSEA, also exhibited similar patterns in Euclidean distances, as larger differences were observed with smaller sample sizes (Fig. 2B). Results from MDT, however, showed noticeable instability over the full range of sample sizes as evidenced by the changes in Euclidean distance, in contrast to the correlation coefficients that remained high (Fig. 2A and B). Moreover, subtle changes in scores were also detected with AUCell and ORA in their Euclidean distances (Fig. 2B).

Given the observation of shifting scores due to sample size differences for the aforementioned approaches, we assessed the impact on the downstream sample clustering. First, as the different clustering algorithms and distance metrics may impact the clustering results, we evaluated the cluster stability with the complete PRECISESADS dataset using different combinations of clustering algorithms and distance metrics. KM or PAM with Euclidean distance yielded the most stable results across all the scoring methods, while hierarchical clustering (hc) with cosine distance performed poorly (Supplementary Fig. S2). Thus, next, K-mean clustering with gene set scores obtained from subsets of the PRECISESADS dataset with different sample sizes was performed. The cluster assignments from each subset were compared with those from the full dataset using failure rates that measured the percentage of mis-assigned samples in a small cohort. Sample clustering using scores from AUCell and ORA yielded highly consistent results regardless of sample sizes, as evidenced by extremely low failure rates across the full range of sample sizes tested (Fig. 2C). GSVA, Z-score, and PLAGE had an inverse relationship between the failure rates and sample sizes, as high failure rates were observed at smaller sample sizes, which then decreased as the sample size increased (Fig. 2C). ssGSEA exhibited low failure rates (≤10%) over the range of sample sizes (Fig. 2C). In contrast, MDT had high variability regardless of sample sizes, as ~50% mis-assignments were detected in most cases (Fig. 2C).

### Missing genes impact on score stability

**Figure 3:**
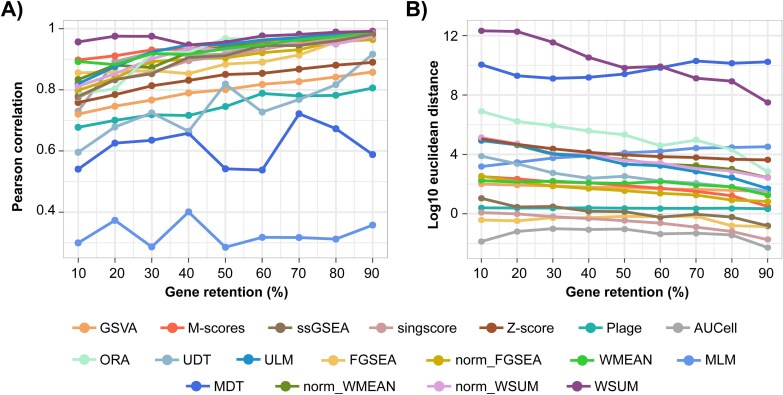
Impact of missing genes on score stability. (A) The average Pearson correlation coefficients and (B) the average Euclidean distance scores on base 10 logarithmic scale computed using subsets of the PRECISESADS dataset retaining variable percentages of genes, ranging from 10% to 90%, compared to scores using the full dataset with 16 919 genes post data pre-processing. Colors denote scoring approaches.

Missing genes in gene expression datasets can be caused by differences in gene expression profiling platforms or biological and technical factors and may therefore introduce additional score variabilities. Thus, we evaluated the impact of missing genes on score stability by randomly removing different percentages of genes from the PRECISESADS dataset and comparing the resulting scores with those obtained using the full dataset. For most scoring approaches, Pearson correlation coefficients remained above 0.75 even with as low as 10% of genes retained (169 genes) in the gene expression matrix (Fig. 3A). The scores were largely unimpacted by a limited number of input genes. This finding was further supported by minimal Euclidean distances detected across most approaches over the range of gene removal tested (Fig. 3B), indicating minimal impact on downstream cross-sample comparisons. However, MLM and MDT were highly sensitive to missing genes, yielding low correlation coefficients (i.e. close to or lower than 0.5) over the full range of gene removal (Fig. 3A), along with high Euclidean distances for MDT (Fig. 3B). Similarly, but to a lesser degree, UDT and PLAGE resulted in moderate correlations at low gene retention percentages (Fig. 3A). Moreover, WSUM showed a drastic increase in Euclidean distance as missing genes increased (Fig. 3B).

### Gene set characteristics impact on score stability

**Figure 4:**
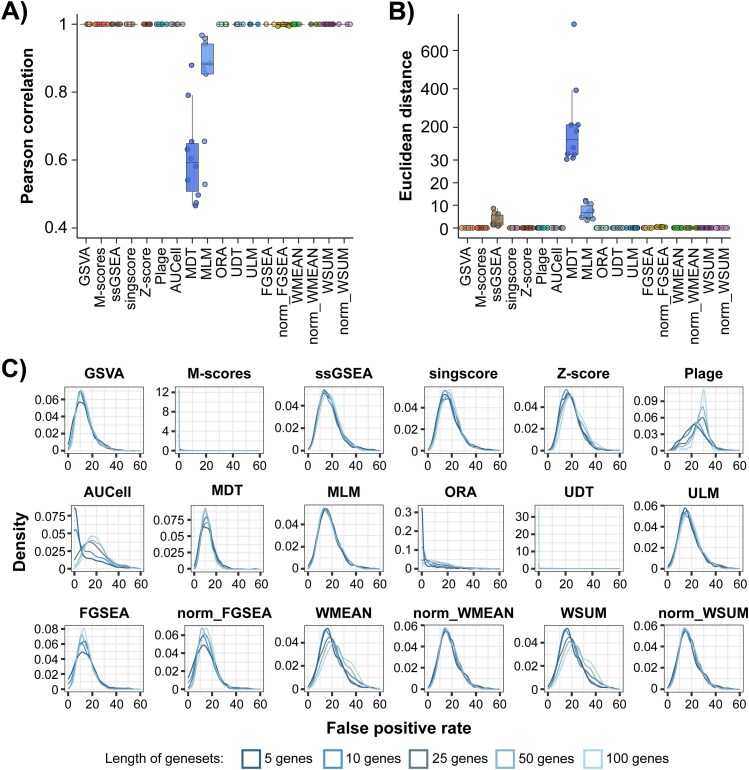
Gene set characteristics impact on score stability. (A) The average Pearson correlation coefficients and (B) the average Euclidean distances comparing scores computed with subsets of the reference gene set database containing different numbers of gene sets, ranging from 1 to 1000, compared to scores computed with the full database containing 4300 gene sets. The average was taken across 10 sampling iterations. Colors denote scoring approaches. (C) The false positive rate (i.e. the percentage of SLE samples with significantly different scores from NHVs among 100 randomly selected SLE samples from the PRECISESADS dataset for a given gene set in a given gene set database) distributions across five arbitrary gene set databases, each containing 1000 gene sets with variable numbers of randomly selected genes, ranging from 5 to 100. Colors denote different gene set databases with different number of genes per gene set.

Many publicly available gene set databases provide comprehensive coverage of biological processes, and cell type-specific functions for scoring, while signatures derived from publications continue to contribute to more targeted functions relevant to specific biological context. The goal of gene set scoring also often ranges from holistic search to targeted query of specific functions of interest. Thus, the impact of the number of gene sets on score stability was evaluated by comparing scores obtained with different numbers of gene sets in each scoring iteration. Our analysis revealed that, while most approaches were unimpacted, MDT and MLM scores were strongly affected by variable gene set numbers, based on both the Pearson correlation and Euclidean distance metrics (Fig. 4A and B), while ssGSEA was also slightly impacted on Euclidean distance (Fig. 4B).

Another consideration when choosing the gene set collection is the accuracy and reliability of gene set annotations. Sources of gene sets can vary from curated and peer-reviewed pathway databases to user provided signatures retrieved from individual publications or experiments. Thus, imprecisions in gene set annotations can be a concern for gene set-based scoring. The sensitivity of scores generated with different approaches were therefore assessed by creating an arbitrary collection of gene sets with randomly selected genes. Those arbitrary gene sets were created with different number of genes to account for potential impacts of the size of a gene set on scores. We observed that the FPR ranged from 10% to 20% for most approaches, with a slightly higher rate detected in PLAGE and lower rates in M-scores, UDT, ORA, and MDT, where the first two approaches (i.e. M-scores and UDT) seemed to be particularly robust against false positives gene sets (Fig. 4C). However, this trend in UDT may be attributed to a large number of pathways with a score as zero. Additionally, Z-score, WMEAN, WSUM, and particularly AUCell, ORA, and PLAGE, had increased FPR as the number of genes per gene set increased (Fig. 4C), suggesting increased effects on score stability with increased false positives gene set memberships.

### Data integration gene set score performance

**Figure 5:**
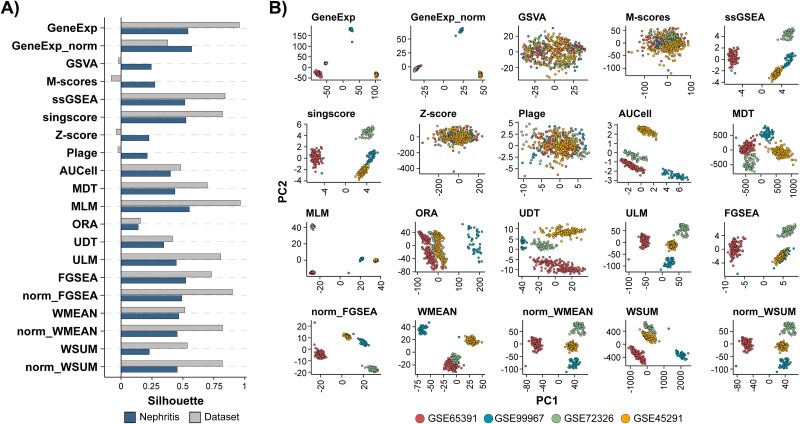
Data integration gene set score performance. (A) Silhouette coefficients computed using four nephritis datasets to assess the impact of the nephritis status and the dataset variables on the sample aggregation distribution based on the corresponding variable categories. Colors denote the two variables described. (B) PCA showing sample profile similarity across the same four nephritis datasets for different scoring approaches and gene expression data. Dots represent samples. Colors denote datasets. GeneExp: log transformed gene expression data; GeneExp_norm: log transformed gene expression data with additional sample-wise z-score normalization.

Comparability of scores from different studies for data integration remains untested. Thus, the performance of gene set scores in cross-study integration using four SLE datasets including SLE patients with or without active pLN or NoLN and healthy volunteers was assessed. These datasets allowed us to test not only the mitigation of study-driven gene set score differences across datasets, but also the preservation of biological signals—nephritis status—during integration. We employed silhouette coefficients to quantitatively assess the association between sample grouping patterns and studies or nephritis status. Specifically, a silhouette coefficient close to 1 or −1 suggests a strong association, while 0 suggests no association. Silhouette coefficients indicate that differences between studies were the major driver of sample grouping patterns across most scoring approaches (Fig. 5A). In contrast, GSVA, M-scores, PLAGE, and Z-score were able to successfully mitigate the impact of differences driven by datasets while preserving sample differentiation on the nephritis status, as their silhouette coefficients for dataset origins were close to 0 while those corresponding to the nephritis status were positive (Fig. 5A). It is worth noting that gene expression data with additional sample-wise scaling, a previously proposed approach to improve reproducibility and mitigate differences among studies during data integration with ML [31, 32], reduced the impact of dataset origin and preserved the contribution of nephritis status, when compared to gene expression data alone (Fig. 5A). However, it still failed to completely mitigate dataset differences. The PCA plots provided additional lines of evidence by delineating how the factors related to datasets and disease states impacted sample distribution across the four datasets, highlighting the effective integration when using scores from GSVA, M-scores, PLAGE, and Z-score (Fig. 5B and Supplementary Fig. S3).

### Across studies score-based machine learning model performance

Downstream applications of gene set scores often include building ML models to predict biological phenotypes [33, 34], such as disease states or treatment responses. However, those models frequently fail in independent validation studies, limiting their application. Thus, the performance of gene set scores obtained with different scoring approaches in ML models were used to predict the pLN status using four SLE datasets from pLN and NoLN patients, two for training and validation and the other two for testing purposes. We observed that norm_WSUM, M-scores, and AUCell achieved overall best performances with the testing datasets based on all used metrics. Indeed, the median MCC and balanced accuracies were above 0.30 and 0.65, respectively (Table 1). Z-score, norm_WMEAN, GSVA, and PLAGE also demonstrated good to moderate performances, with the median MCC higher than 0.20 and balanced accuracy above 0.60 (Table 1). Conversely, other scoring approaches and the direct use of gene expression data, including the sample-wise normalized version, showed suboptimal performance (Table 1).

**Table 1:** Performance of score-based ML models to predict pLN^a^^,^^b^

	**MCC**	**BAcc**	**Prec**	**Recall**	**Spec**	**FScore**	**NPV**
**norm_WSUM**	0.36 (±0.15)	0.68 (±0.08)	0.55 (±0.33)	0.47 (±0.14)	0.95 (±0.14)	0.43 (±0.17)	0.76 (±0.24)
**M-Scores**	0.3 (±0.22)	0.69 (±0.1)	0.53 (±0.44)	0.72 (±0.12)	0.76 (±0.29)	0.43 (±0.27)	0.78 (±0.22)
**AUCell**	0.28 (±0.2)	0.67 (±0.08)	0.52 (±0.42)	0.7 (±0.18)	0.75 (±0.2)	0.33 (±0.26)	0.83 (±0.22)
**norm_WMEAN**	0.27 (±0.17)	0.63 (±0.09)	0.33 (±0.4)	0.3 (±0.2)	0.96 (±0.05)	0.38 (±0.22)	0.72 (±0.26)
**Z-score**	0.24 (±0.25)	0.71 (±0.12)	0.41 (±0.38)	0.8 (±0.1)	0.64 (±0.32)	0.53 (±0.31)	0.73 (±0.37)
**Plage**	0.23 (±0.09)	0.66 (±0.05)	0.47 (±0.37)	0.7 (±0.24)	0.69 (±0.17)	0.28 (±0.19)	0.76 (±0.27)
**GSVA**	0.21 (±0.11)	0.68 (±0.07)	0.4 (±0.32)	0.7 (±0.14)	0.66 (±0.05)	0.37 (±0.21)	0.76 (±0.27)
**ULM**	0.2 (±0.14)	0.6 (±0.08)	0.49 (±0.3)	0.51 (±0.29)	0.84 (±0.29)	0.51 (±0.27)	0.71 (±0.32)
**MDT**	0.19 (±0.13)	0.58 (±0.07)	0.41 (±0.41)	0.62 (±0.36)	0.62 (±0.32)	0.24 (±0.27)	0.73 (±0.25)
**WMEAN**	0.18 (±0.14)	0.61 (±0.07)	0.58 (±0.29)	0.38 (±0.25)	0.86 (±0.19)	0.36 (±0.21)	0.74 (±0.26)
**ssGSEA**	0.16 (±0.18)	0.56 (±0.09)	0.69 (±0.42)	0.17 (±0.17)	0.98 (±0.03)	0.19 (±0.23)	0.75 (±0.26)
**singscore**	0.16 (±0.13)	0.54 (±0.05)	0.22 (±0.47)	0.09 (±0.12)	1 (±0.03)	0.16 (±0.16)	0.68 (±0.28)
**ORA**	0.15 (±0.2)	0.56 (±0.11)	0.35 (±0.35)	0.72 (±0.29)	0.43 (±0.28)	0.39 (±0.3)	0.91 (±0.28)
**UDT**	0.14 (±0.08)	0.57 (±0.06)	0.42 (±0.32)	0.5 (±0.28)	0.69 (±0.35)	0.27 (±0.27)	0.69 (±0.39)
**WSUM**	0.14 (±0.13)	0.56 (±0.09)	0.34 (±0.28)	0.6 (±0.37)	0.68 (±0.41)	0.28 (±0.31)	0.74 (±0.44)
**norm_FGSEA**	0.13 (±0.16)	0.56 (±0.05)	0.19 (±0.38)	0.17 (±0.29)	0.92 (±0.26)	0.17 (±0.09)	0.66 (±0.31)
**geneExp**	0.03 (±0.1)	0.52 (±0.06)	0.37 (±0.32)	0.51 (±0.3)	0.56 (±0.31)	0.4 (±0.33)	0.7 (±0.42)
**geneExp_norm**	0.01 (±0.17)	0.5 (±0.1)	0.4 (±0.37)	0.96 (±0.26)	0.06 (±0.38)	0.39 (±0.29)	0.48 (±0.45)
**MLM**	0 (±0.05)	0.5 (±0.04)	0.11 (±0.29)	0.38 (±0.47)	0.8 (±0.49)	0.17 (±0.35)	0.36 (±0.44)
**FGSEA**	0 (±0.11)	0.5 (±0.07)	0 (±0.31)	0 (±0.18)	1 (±0.05)	0 (±0.12)	0.65 (±0.3)

^a^The median of metric performances (±SD) across iterations is shown. Table summarizing performance metrics of machine learning models using gene set scores to predict lupus nephritis.

^b^BAcc, balanced accuracy; Prec, precision; Spec, specificity; NPV, negative predictive value.

### Association between molecular scores and clinical features

**Figure 6:**
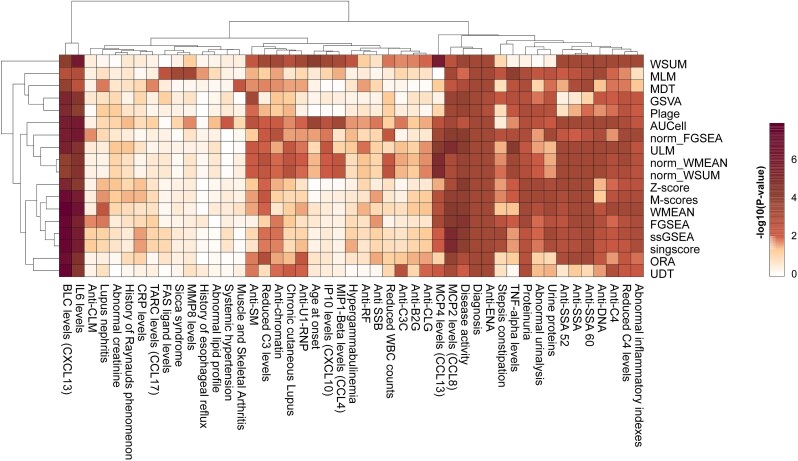
Association between molecular scores and clinical features. The heatmap shows the minus log10 transformed *P*-values from enrichment analyses between a given clinical feature (columns) and sample clusters generated with scores from different scoring approaches (rows).

To link molecular signatures to clinical manifestations, the implementation of gene set scores for patient stratification and subsequent identification of patient groups with unique clinical characteristics was assessed. Patients in the PRECISESADS dataset were clustered using gene set scores from the different scoring methods described above. Enrichment analysis of 67 clinical features across the resulting clusters was run to determine the association between the distribution of clinical features and patient groups. Those clinical features captured distinct aspects of disease states, ranging from disease activity, symptoms, and organ-specific manifestations to laboratory measurements of systemic biomarkers, such as the levels of autoantibodies or serum cytokines. All scoring approaches were able to successfully link molecular signatures to clinical-relevant disease states, particularly those expected to be related to SLE, indicated by strong enrichments across patient groups and consistent patterns (Fig. 6). To further determine whether gene set scores can yield clinical meaningful association, we also assessed the association between gene set scores from M-Scores in the PRECISESADS cohort and two key clinical features in SLE, including the global disease activity defined in PRECISESADS [17] and the presence of pLN. Among the top 20 gene sets most significantly associated with disease activity, we identified pathways related to apoptosis, pyroptosis, interferon (IFN) gamma signaling, and protein localization to the membrane. In contrast, gene sets linked to neutrophils, including neutrophil degranulation and chemotaxis, were most significantly enriched in patients with pLN. These results are in line with current understanding of molecular disease mechanism, supporting applications of gene set scores for clinical feature association analyses.

## Discussion

In this work, we systematically benchmarked the performance of 18 single-sample scoring approaches on transcriptome data. We evaluated a comprehensive range of commonly encountered scenarios, when applying the scoring approach to datasets with different characteristics or for different downstream applications. The results revealed different features of the scoring approaches, including their sensitivity and robustness against suboptimal input data, providing a data-based framework when choosing the most appropriate approach for a given task. Major applications of single-sample gene set scores for patient stratification, ML, and data integration were also assessed, shedding light on the broader and more strategic implementations for precision medicine research.

The performance of different scoring approaches in a variety of scenarios with input data limitations affecting score stability were methodically evaluated. Table 2 includes a brief overview of algorithm principles, summary of the main results from each test and practical recommendations on the best use cases given the characteristics of the input data and the analytical goals. The characteristics of the gene expression data and the gene set database can differ from study to study and depend on the goal of the study, making their use for other applications difficult. When handling several main characteristics assessed in this work, including sample size, missing genes in the gene expression matrix and the number of gene sets in the gene set database, some scoring approaches were more sensitive to certain characteristics than others and produced more variable results when handling edge cases. For example, MDT, PLAGE, GSVA, Z-score, and ssGSEA were unstable with small sample sizes, reducing reproducibility when comparing scores with those from larger cohorts. This observation may be attributed to their underlying algorithm, as those approaches require cross-sample statistics, such as variance between samples, which can become noisy in small cohorts leading to reduced reproducibility. Score stability for MDT, MLM, and WSUM was impacted by missing genes in the gene expression data, a challenge when analyzing data generated from different assays or platforms, or with different technical qualities. This observation can also be explained by the statistical principles underlying each algorithm. PLAGE relies on SVD of gene expression within each gene set, which becomes unstable as the number of contributing genes decreases. Similarly, multivariate model-based approaches (i.e. MLM, MDT, UDT) also can be substantially impacted by missing genes, as their estimation is based on the joint contribution of all the gene members in a gene set including their relationships. In contrast, enrichment-based approaches, such as GSVA, FGSEA, and ssGSEA, as well as descriptive statistics-based approaches, such as M-scores and Z-scores, are less dependent on gene set completeness and therefore remain more robust to missing genes. In the case of WSUM, the observed higher variability in Euclidean distance likely reflects its direct dependence on the number of genes within each gene set. These reported differences suggest that model-based scores may be better suited when analyzing high-dimensional, well-annotated transcriptome data of high quality, whereas enrichment-based methods are suitable when gene coverage is limited, such as with data from targeted panels or microarrays. In terms of the number of gene sets for scoring, most approaches, except for MDT, MLM, and, to a much lesser extent, ssGSEA, were not sensitive to the differences. The reason is that affected approaches normalize or scale their scores relative to the entire gene set universe analyzed, rather than computing them in an absolute, self-contained manner for each gene set. Only M-scores and UDT showed robustness against inaccurate gene set annotations. Those factors are particularly relevant when testing a hypothesis derived from a particular publication or study [35]. Given our results, careful considerations should be taken when handling data with aforementioned limitations, simultaneously analyzing scores from datasets with different numbers of genes or comparing scores for a given gene set computed with the gene set alone or the entire gene set database. Given our results, careful considerations should be taken when handling data with aforementioned limitations, simultaneously analyzing scores from datasets with different numbers of genes or comparing scores for a given gene set computed with the gene set alone or the entire gene set database.

**Table 2:** Summary of benchmark results and recommendations^a^

**Method**	**Algorithm principle**	**Runtime**	**Robustness to**				**Data integration**	**ML performance**	**Recommendations**
			**Sample size**	**Missing genes**	**Gene set database size**	**Gene set database quality**			
**GSVA**	Rank-based enrichment	Low	No	High	High	Moderate	Yes	Moderate	Integration/ML modeling with large cohorts. Not suitable for comparisons between small cohorts
**M-Scores**	Distribution-based	Moderate	Yes	High	High	High	Yes	High	Recommended when reference samples are available
**ssGSEA**	Rank-based enrichment	Low	No	High	Moderate	Moderate	No	Low	Individual/functional studies, large cohorts.
**singscore**	Rank-based enrichment	Low	Yes	High	High	Moderate	No	Low	Individual/functional studies
**Z-scores**	Distribution-based	Low	No	High	High	Moderate	Yes	Moderate	Integration/ML modeling with large cohorts. Not suitable for comparisons between small cohorts
**Plage**	SVD	Low	No	Low	High	Low	Yes	Moderate	Integration/ML modeling with large cohorts. Not for comparisons between small cohorts. Similar cohort characteristics required (i.e. platforms)
**AUCell**	Rank-based enrichment	Low	Yes	High	High	Moderate	No	High	Individual/functional studies and ML modeling
**ORA**	Based on Fisher’ exact test	Moderate	Yes	Moderate	High	High	No	Low	Individual/functional studies
**UDT**	Based on decision trees	High	Yes	Low	High	High	No	Low	Individual/functional studies. Preferably with well-characterized gene sets (weighted networks)
**MDT**	Based on decision trees	High	No	No	No	High	No	Low	Individual/functional studies. Preferably with well-characterized gene sets (weighted networks). Not for comparisons between small cohorts
**ULM**	Based on linear model	High	Yes	High	High	Moderate	No	Low	Individual/functional studies. Preferably with well-characterized gene sets (weighted networks)
**MLM**	Based on linear model	High	Yes	No	No	Moderate	No	Low	Individual/functional studies. Preferably with well-characterized gene sets (weighted networks)
**FGSEA**	Rank-based enrichment	Moderate	Yes	High	High	Moderate	No	Low	Individual/functional studies
**norm_FGSEA**	Rank-based enrichment	High	Yes	High	High	Moderate	No	Low	Individual/functional studies
**WMEAN**	Aggregation-based	Low	Yes	High	High	Low	No	Low	Individual/functional studies
**norm_WMEAN**	Aggregation-based	Low	Yes	High	High	Moderate	No	High	Individual/functional studies
**WSUM**	Aggregation-based	Low	Yes	No	High	Low	No	Low	Individual/functional studies. Similar cohort characteristics required
**norm_WSUM**	Aggregation-based	Low	Yes	High	High	Moderate	No	High	Individual/functional studies

^a^The table summarizes the performance and characteristics of the single-sample gene set scoring approaches evaluated in this study across several scenarios. Columns include a brief summary of the algorithm principle, performance (including runtime, robustness to different sample size, impact of missing genes, gene set database size, and quality on score, data integration, and ML performance), and practical recommendations on the best use cases. Individual studies indicate single cohort analysis. Functional studies indicate analysis with goals for functional interpretation of gene expression, such as signature enrichment. Table summarizing the performance of all the evaluated single-sample gene set scoring approaches and providing practical recommendations.

Our work also supports broad downstream applications of single-sample gene set scores in patient stratification, predictive modeling of clinical states, and data integration. Besides direct comparison of gene set scores across groups of interest, scores often serve as an intermediate input for downstream applications for patient stratification with molecular-based sample clustering and association with clinical features. They hold potential as an alternative strategy for predictive modeling of disease statues or treatment response [36]. Indeed, most approaches successfully revealed associations between molecular patterns across patient groups and disease-relevant clinical features. However, broader applications are limited by reproducibility of resulting models or observations across studies. Most published models failed to be validated in independent cohorts, with a few exceptions in cancer research when data comparability was addressed across studies by focusing on a pre-selected list of genes and samples or using gene-wise normalization [31, 32, 37, 38]. Lack of understanding of the advantages and limitations of different scores for predictive modeling hinders their broader application. Our results yielded different performances across the different approaches, among which norm_WSUM, M-scores, and AUCell ranked as top approaches according to multiple performance metrics and testing iterations. Others such as norm_WMEAN, Z-score, PLAGE, and GSVA also achieved moderate performances. Although further testing under different diseases or biological contexts will be needed to generalize these observations, the results provided evidence of a significant improvement on performance when considering scoring approaches for predictive modeling instead of gene-expression based models. Moreover, M-scores, GSVA, Z-score, and PLAGE effectively mitigated dataset-driven differences without the need for additional data processing, while preserving biological signals of interest, making them suitable for data integration. These observations may be attributed to their intrinsic ability to harmonize the data across cohorts, particularly whether it can effectively project heterogeneous datasets into a comparable space. Among the scoring approaches with better performance, GSVA, Z-score, and PLAGE apply transformations based on rank distributions, which mitigate cohort specific biases while preserving biologically meaningful variation. Similarly, M-scores normalizes the data using a set of reference samples within each dataset, which allows the scores to be harmonized to a comparable normal distribution. On the other hand, some approaches performed well in modeling but failed in data integration, including AUCell, norm_WSUM, and norm_WMEAN. For AUCell, by evaluating whether the top-ranked genes in the samples are enriched for a given gene set, it minimizes the dependence on the raw expression scale, which facilitates better alignment across cohorts even without explicit normalization. Similarly, norm_WSUM and norm_WMEAN were able to capture consistent within-cohort gene set activity patterns. These relative differences are highly informative for supervised learning tasks, which explains their strong performance in predictive modeling despite limited utility for direct data integration.

Our benchmark demonstrated consistent performance between scoring approaches sharing similar underlying algorithms, with small variation within each method group due to subtle technical differences, while larger differences across method groups were observed. Differences in their underlying algorithms have profound impacts on their sensitivity to different characteristics of input data and unbiased preservation of biological heterogeneity. While some disease-specific aspects may differ, the technical conclusions of our work are expected to be generalizable across diseases. Distribution-based methods, such as M-scores, Z-score, and PLAGE, demonstrated excellent overall performance, regardless of the presence of missing genes and the size of the gene set database, and enabled effective data integration. M-scores also demonstrated top performance in predictive modeling and robustness against false positive results. It is worth noting that, to achieve the optimal performance with M-scores, the use of NHV data as reference is required as described in the original method [5]. However, Z-score and PLAGE are impacted by small sample sizes, as their data scaling relies on all the samples in the cohort. Like distribution-based methods, rank-based methods generally demonstrate great performances under many scenarios. Particularly, GSVA enabled data integration and showed moderate performance in predictive modeling. Although it was impacted by small sample sizes, it can be considered for large cohorts, similarly to Z-score. Rank-based approaches are less suitable for contrast analysis between groups, such as case-control differential analysis. Due to the rank transformation, these approaches do not take into account of the magnitude differences in expression, which can skew the variability and trend when the actual differences are small, subsequently increasing the likelihood of false positives (as shown in Fig. 4B). Similarly, the composition-dependent distortions introduced by ranking can limit the identification of moderate signals and thus the ability to represent continuous phenotypes, such as pseudo-time trajectories or quantitative gradients like cellular states, where preserving magnitude and variance structure is essential for accurate inference. Conversely, these methods can be highly valuable for detecting shared patterns across independent studies and for highlighting intra-group variability, making them particularly useful for clustering. Additionally, other methods were impacted under different scenarios and showed limited applications with data integration or moderate to low performance in predictive modeling, except for AUCell, norm_WMEAN, norm_WSUM, and WMEAN with top performance in modeling, suggesting their potential in model-based applications with suitable datasets.

Our case study on molecular and clinical associations using gene set scores further demonstrated the downstream application of molecular scores, linking to clinical manifestations. Most scoring methods can effectively reveal enrichment of certain clinical features in patient subpopulations, indicating aligned underlying molecular mechanisms and selected clinical manifestations (Fig. 6). For example, several cytokines, such as CXCL13, CCL8, and TNF, which relate to B-cell and lymphoid structure biology, monocyte/macrophage recruitment, and classical pro-inflammatory cytokine signaling, diagnostic auto-antibodies, such as anti-SSA, anti-dsDNA, and anti-C4, as well as disease activity and urinalysis abnormalities consistent with lupus nephritis, strongly segregate individuals, being significantly associated to patient clusters across approaches. In contrast, several other clinical features, such as cytokines including CCL17, CXCL10, and CCL4 that are more closely associated with T-cell–driven chemotaxis, interferon/Th1 responses, and selective monocyte/eosinophil recruitment, as well as anti-RF, showed limited associated with the dominant axes of molecular variation underlying patient clustering. Further analyses are warranted to explain the biological trends which are behind the performances of these analytical methods.

Enabling the application of knowledge from gene–gene interaction networks for gene set scoring emerged as an option in many recently developed scoring approaches (e.g. AUCell, MDT, MLM, ORA, UDT, ULM, FGSEA, norm_FGSEA, WMEAN, norm_WMEAN, WSUM, and norm_WSUM), in contrast to commonly used approaches (e.g. M-scores, GSVA, ssGSEA, Z-score, singscores, and PLAGE) relying purely on gene sets. Those network-enabled approaches take into consideration the interaction weight between genes in the same gene set or pathway, retrieved from experimental validation, and thus can increase the confidence in functional data transformation. However, gaps in knowledge regarding validated gene–gene interactions, including the weights and directionalities, and in broader disease contexts rather than limited diseases, significantly limit their broader application [39]. However, these methods hold the potential to provide additional biological insights beyond transcriptome alone, when gene–gene interaction data with high confidence is readily available to support well characterized pathways [15]. Thus, their application should be guided by the availability and quality of relevant network information to avoid bias in findings and interpretations due to suboptimal network knowledge. Similarly, future work to advance gene set scoring approaches can focus on incorporating additional evidence from different sources of data types, such as omics data, as demonstrated in a few initial studies [40, 41]. It holds the potential to further improve the translation of high-dimensional molecular data to functionally interpretable metrics.

**Key Points**

- Single-sample gene set scoring enables functional interpretation of transcriptomic data while preserving patient-specific variability, essential for precision medicine research.
- The stability and reproducibility of scoring methods can be significantly affected by factors such as sample size, missing genes, and gene set characteristics.
- These scoring approaches enhance downstream applications like patient clustering, clinical feature association, and disease state prediction using machine learning.
- Benchmarking across diverse scenarios provides guidance for selecting appropriate scoring methods based on data quality and research objectives.

## Supplementary Material

Supplementary_materials_bbaf684_Figure1

Supplementary_materials_bbaf684_Figure2

Supplementary_materials_bbaf684_Figure3

Supplementary_materials_bbaf684

## Data Availability

SLE Datasets with pLN samples used in this study were downloaded from the GEO database under GSE65391, GSE99967, GSE72326, and GSE45291. The PRECISESADs dataset can be accessible upon request [17]. The code used to run benchmark analyses is available at https://github.com/dtordom/MolecularScoringBenchmark.
