# Supplementary figures and images for: Benchmarking single-sample gene set scoring methods for application in precision medicine
Source: Brief Bioinform. 2025 Dec 17;26(6):bbaf684. doi: 10.1093/bib/bbaf684 (PMC12710473; doi:10.1093/bib/bbaf684)

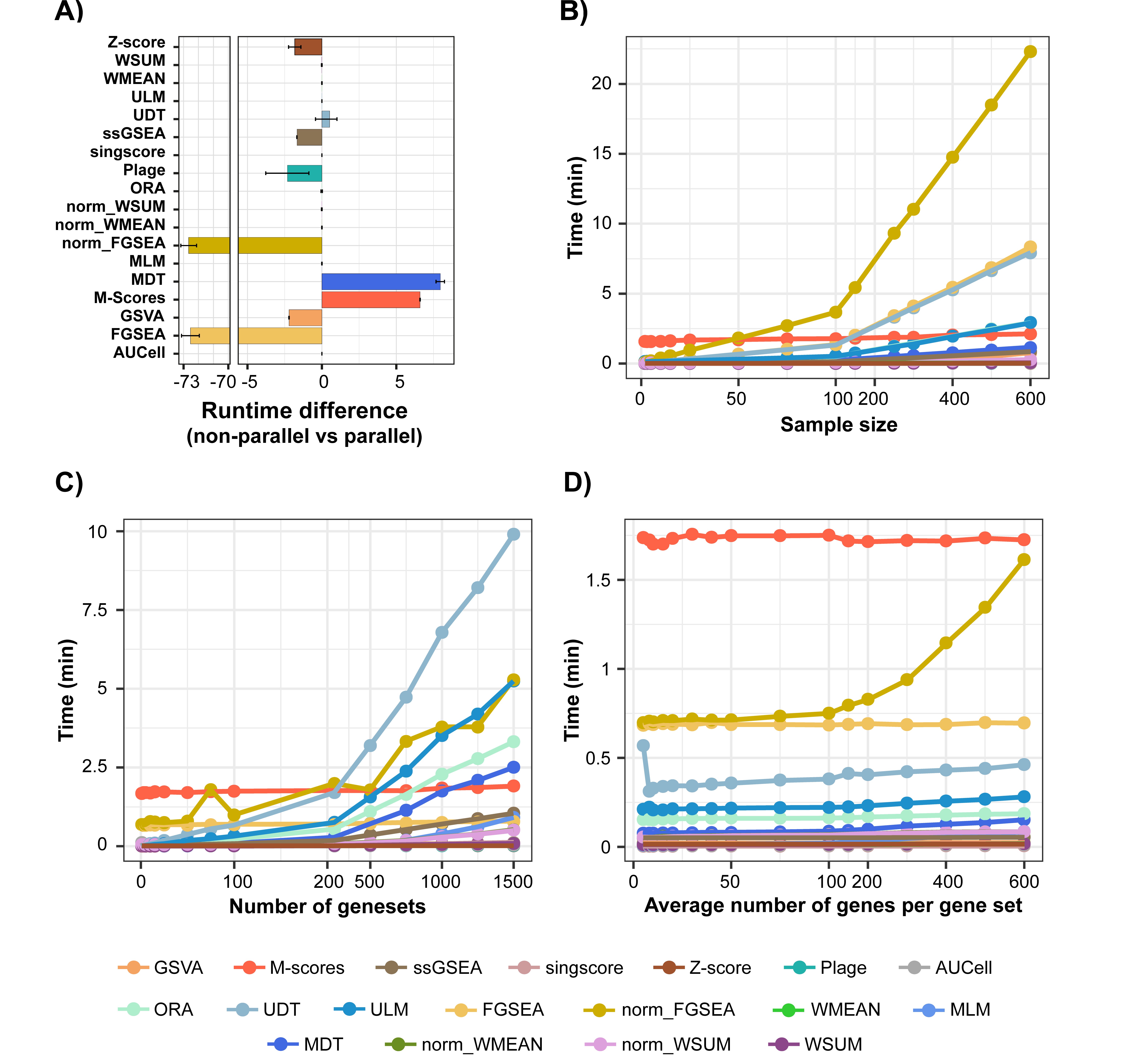

Supplement: Supplementary_materials_bbaf684_Figure1 [file supplementary_materials_bbaf684_figure1.jpeg]

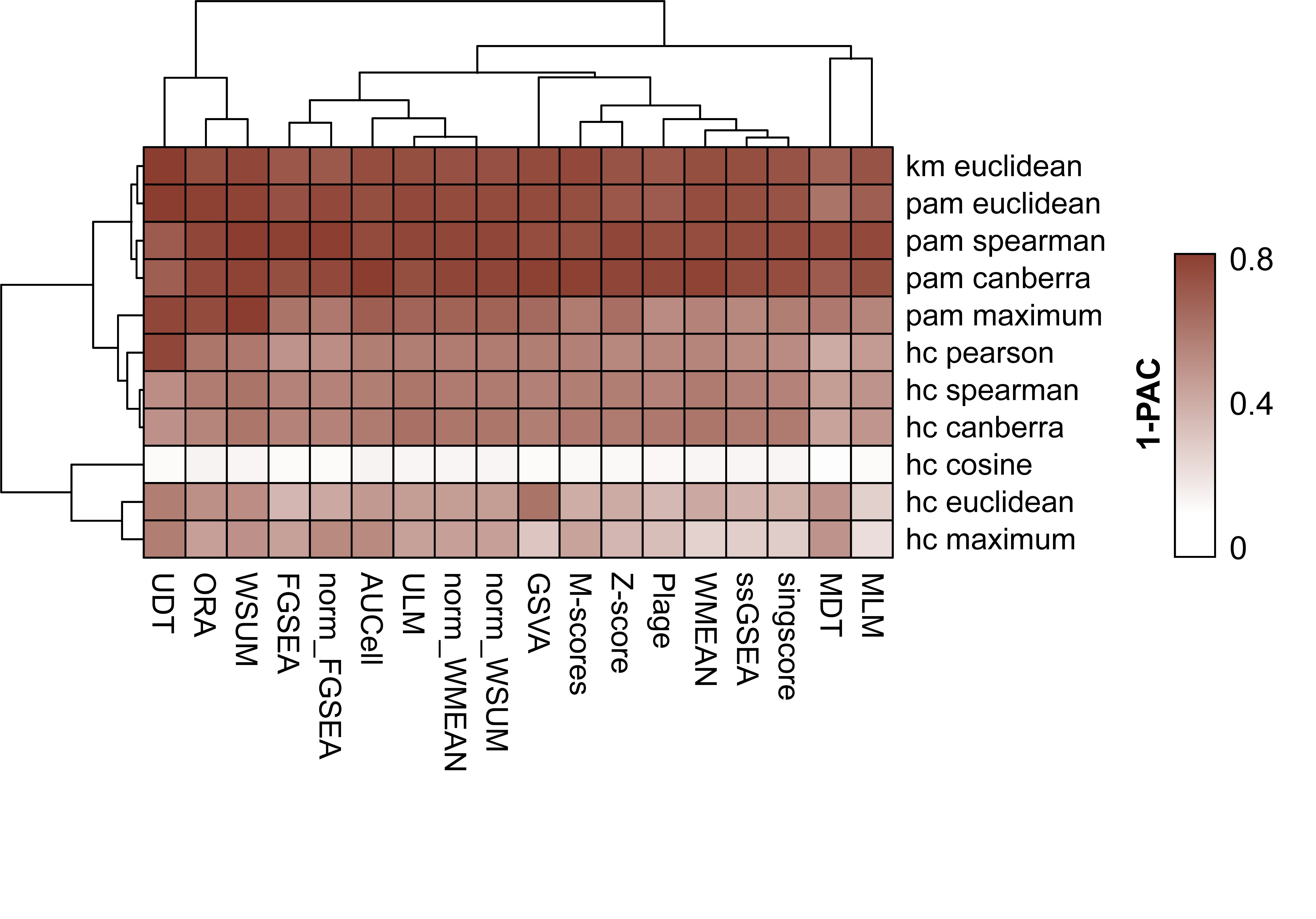

Supplement: Supplementary_materials_bbaf684_Figure2 [file supplementary_materials_bbaf684_figure2.jpeg]

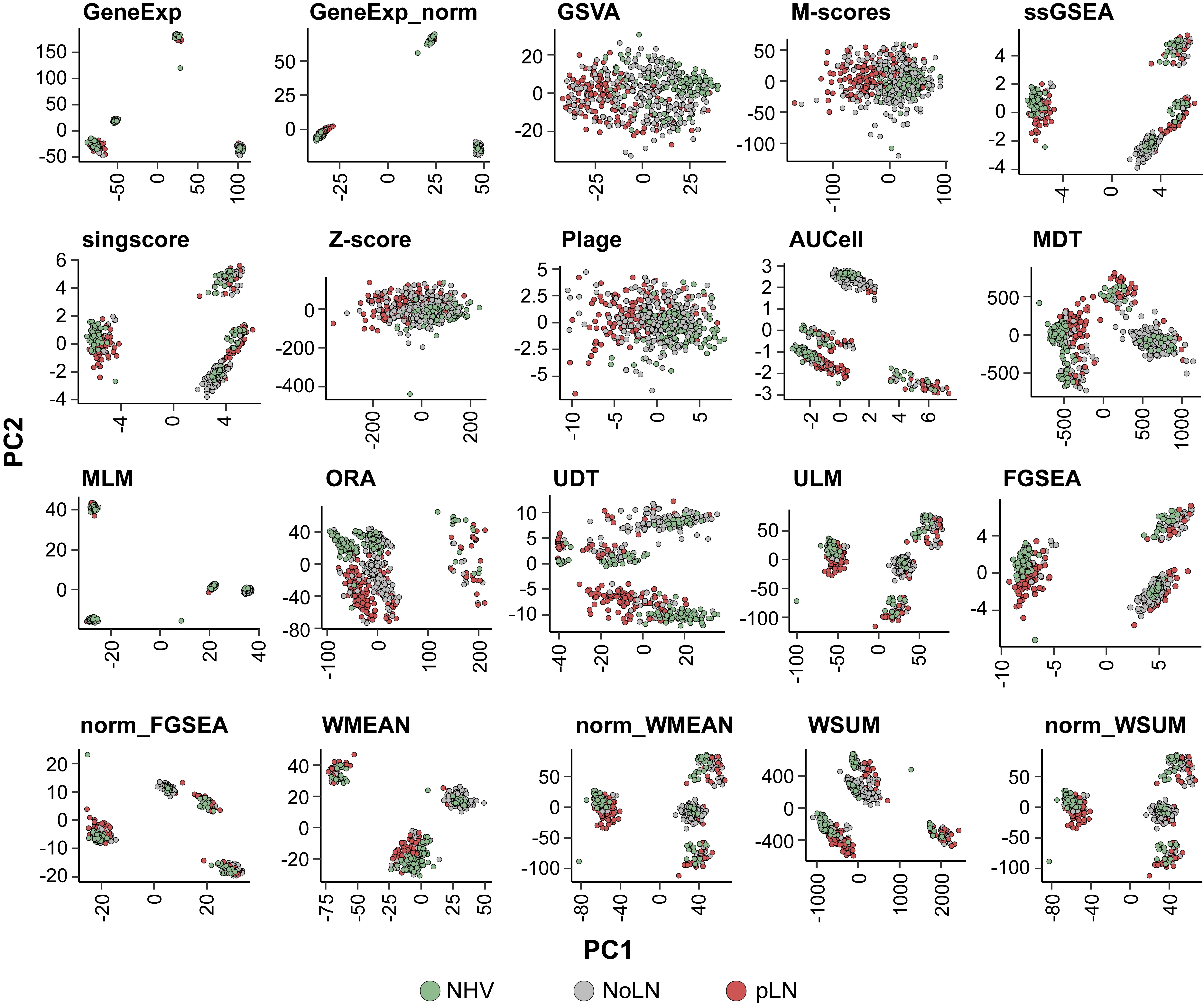

Supplement: Supplementary_materials_bbaf684_Figure3 [file supplementary_materials_bbaf684_figure3.jpeg]

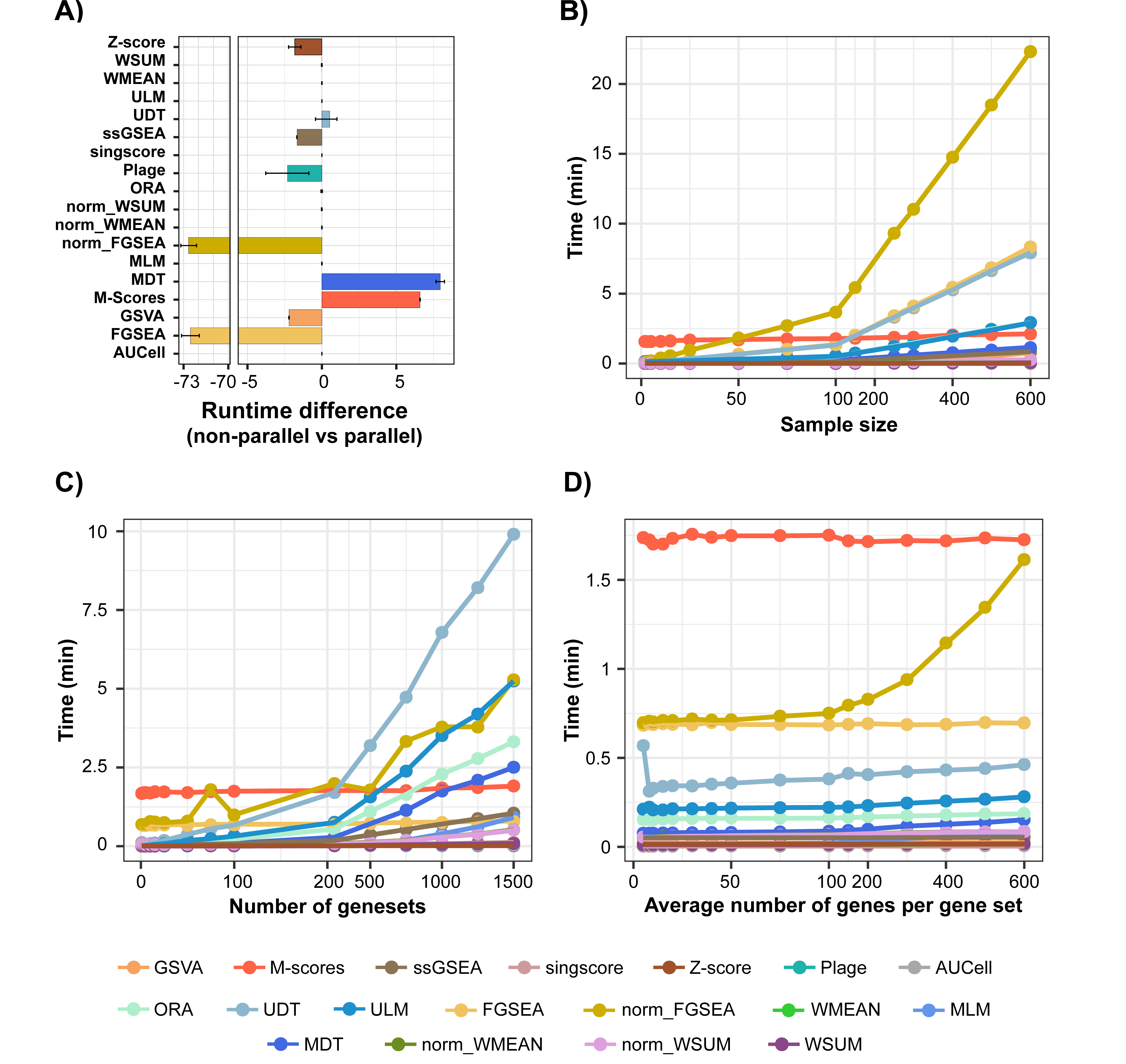

Supplement: Supplementary_materials_bbaf684 [file supplementary_materials_bbaf684.zip › Supplementary_materials_bbaf684_Figure1.tif]

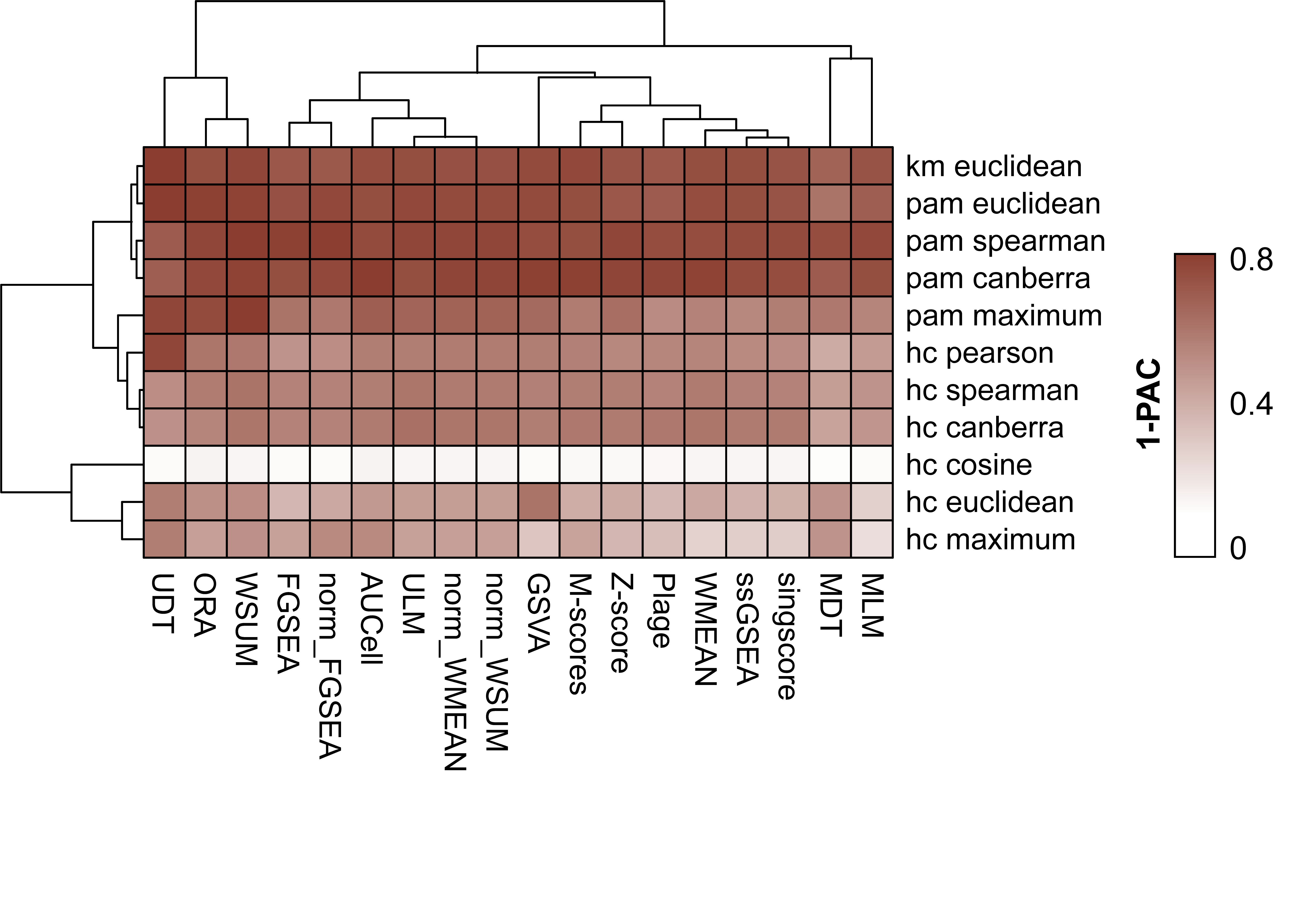

Supplement: Supplementary_materials_bbaf684 [file supplementary_materials_bbaf684.zip › Supplementary_materials_bbaf684_Figure2.tif]

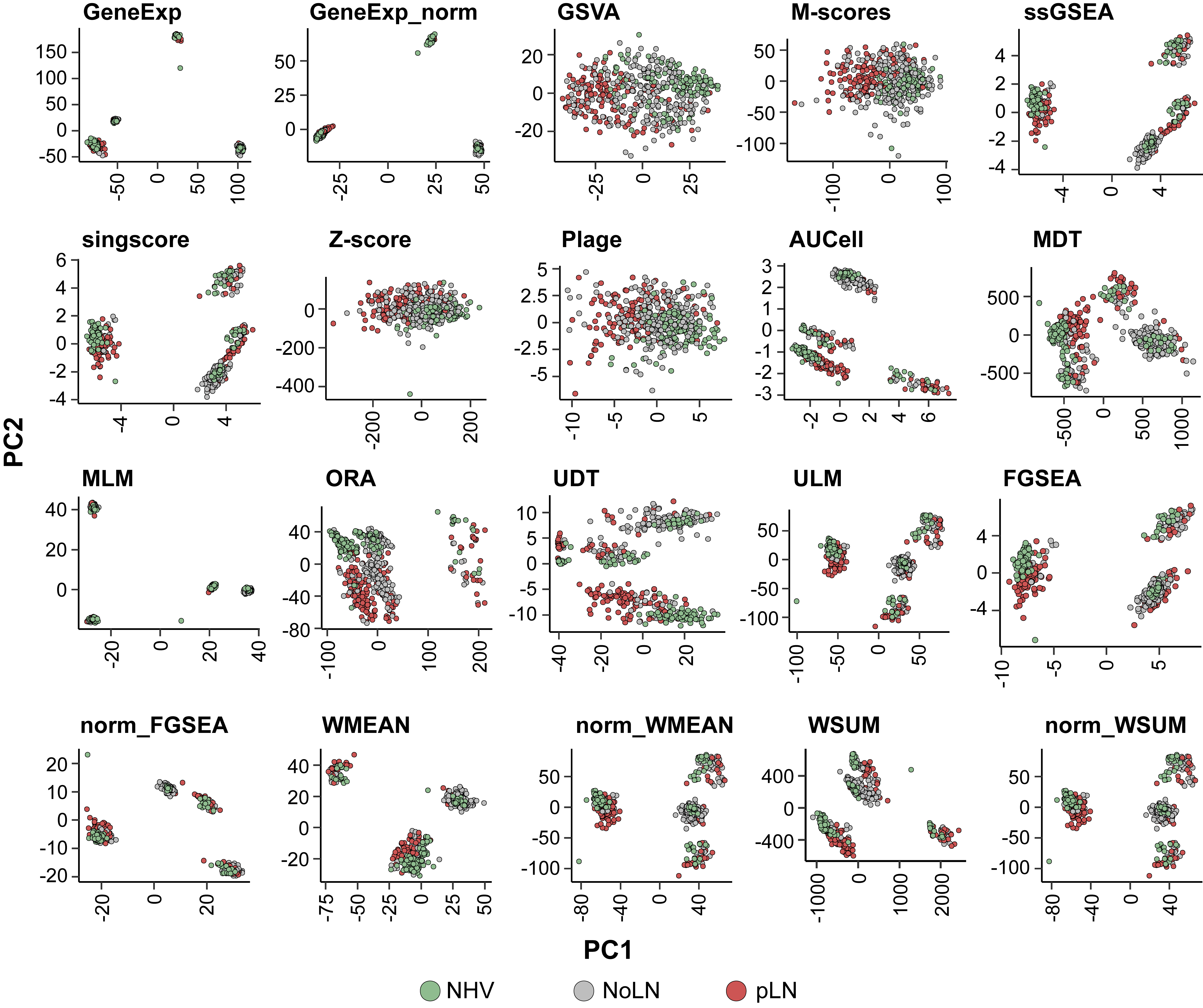

Supplement: Supplementary_materials_bbaf684 [file supplementary_materials_bbaf684.zip › Supplementary_materials_bbaf684_Figure3.tif]
